# Evaluating Remimazolam for Procedural Sedation

**DOI:** 10.2174/011570159X367968250117034551

**Published:** 2025-02-06

**Authors:** Genevieve Monanian, Brandon Yonel, Deborah Rupert, Alina Razak, Michelle DeLemos, Sergio D. Bergese

**Affiliations:** 1 Department of Anesthesiology, Stony Brook University Health Science Center, Stony Brook, NY, 11794-8480, USA;; 2 Renaissance School of Medicine, Stony Brook University, Stony Brook, NY, 11794-8480, USA;; 3 Department of Neurological Surgery, Stony Brook University Health Science Center, Stony Brook, NY, 11794-8480, USA

**Keywords:** Remimazolam, procedural sedation, remimazolam, clinical trials, analgesic properties, depressed consciousness

## Abstract

Remimazolam, a novel short-acting benzodiazepine, has garnered interest in the realm of procedural sedation. Targeting a desired level of sedation requires a medication with both anesthetic and analgesic properties, particularly in non-operating room anesthetizing locations. *Via* gamma-aminobutyric acid type A receptor agonism, Remimazolam has demonstrated organ-independent metabolism and rapid time to both onset and recovery and has efficacy for procedural sedation in clinical trials. This article provides a review of the current literature on the utility of remimazolam for procedural sedation.

## 1 INTRODUCTION

Procedural sedation, as outlined by the American Society of Anesthesiologists Task Force and accepted by the Joint Commission, ranges on a spectrum from anxiolysis to general anesthesia [1]. The level of sedation is determined by the responsiveness of the patient, presence of spontaneous ventilation, need for airway intervention, and impact on cardiovascular function [2]. Moderate sedation (previously “conscious sedation”), most typically used for procedures, specifically requires depressed consciousness wherein the patients may continue to respond purposefully to verbal commands, and the patient is able to ventilate and protect their airway spontaneously. In contrast, deep sedation may require intervention for cardiorespiratory support or the need to convert to general anesthesia, and can result when moderate sedation is improperly targeted. The risk for “incidental” deep sedation with the goal of moderate sedation is higher amongst patients with preexisting medical conditions (such as obstructive sleep apnea, obesity, or epilepsy), in elderly and pediatric populations, and in patients who concomitantly use anxiolytic or psychotropic drugs or have long-term use of benzodiazepines [3-6]. In sum, procedural sedation is the practice of providing pharmacological agents regardless of route with the goal of providing tolerability to patients undergoing invasive diagnostic or surgical therapeutic procedures. That tolerability requires proper pain control, alleviation of anxiety, and often a sedation level (“depth”) that balances the need for suppressed psychomotor activity with that for response to commands.

Procedural sedation and analgesia are typically accomplished by administering multiple classes and combinations of analgesic, sedative, and anesthetic drugs [7]. The American Society of Anesthesiologists Task Force on Moderate Sedation published practice guidelines in 2018 regarding common agents used in combination for procedural sedation, including benzodiazepines, opioids, and dexmedetomidine; however, these agents can potentiate adverse reactions, including excess sedation, respiratory depression, and cardiovascular compromise [2]. Further, part of the complexity of providing procedural sedation is that it is often administered in a variety of clinical settings (non-operating room anesthesia) [8-10] and by a diverse set of practitioners [9, 11-13]. Combined, these variables foster safety concerns and rigorous examination of approaches to minimizing side effects and adverse events [14].

Moreover, the approach to achieving sufficient sedation depends on the setting in which it is administered (for example, in the intensive care unit or for non-operating room anesthesia). Procedural sedation for these environments has unique challenges, including abbreviated case times, high patient volume loads, therefore, high room turnovers, in conjunction with significant pain control needs [15-17]. The growing number and complexity of procedures that benefit from sedation ensures the continued need for the development of sedatives with pharmacological profiles that optimize these patient experiences and their efficiency. Meanwhile, the healthcare consequences, along with the financial impact of adverse events associated with procedural sedation, demand the development of anesthetic agents with better safety profiles, rapid titration to effect, and enhanced recovery [18]. Herein, we highlight the developing role of the latest sedative agent, remimazolam (previously CNS-7056).

Remimazolam is a novel benzodiazepine with an ultra-short duration of action with an approval for intravenous procedural sedation [19]. Remimazolam is among the newer agents introduced for the maintenance of procedural sedation and was approved by the United States Food and Drug Administration and China in 2020, followed by approval by the European Medicines Agency and South Korea in 2021 [20, 21]. Like other drugs in the benzodiazepine class, this short-acting drug is a gamma-aminobutyric acid (GABA) type A receptor agonist that produces central nervous system inhibition [19]. However, the structure of remimazolam differs in the addition of a carboxylic ester moiety to the benzodiazepine core [22]. This addition contributes to the novel drug’s favorable rapid metabolism to a pharmacologically inactive metabolite as it makes it susceptible to hydrolysis by non-specific tissue esterase [19, 22]. In clinical trials, remimazolam was demonstrated to have the previously described short duration of action, low context-sensitive half-time, and shorter, as well as more consistent, recovery [23, 24]. These properties of remimazolam contribute to its characterization as a soft drug, which is defined as a drug that is metabolically active and then rapidly inactivated in the body [23]. The previously described organ-independent metabolism, rapid time to onset and recovery, and low context-sensitive half-time make remimazolam a drug of great interest in the realm of procedural sedation. In this review, we intend to explore the properties of remimazolam as well as its utility in the realm of procedural sedation.

## 2 MECHANISM OF ACTION

Remimazolam is an “ultra” short-acting benzodiazepine that works by binding central gamma-aminobutryic acid (GABA)-A receptors between alpha and gamma subunits in a highly receptor-specific manner [25, 26]. Like other benzodiazepines, remimazolam binds to the extracellular site of the GABA-A receptor, the main inhibitory receptor in the central nervous system [27]. GABA-A receptors are pentameric structures that belong to the “cys-cys loop” of ligand-gated ion channels [28, 29]. When bound by two GABA molecules, these channels undergo a structural, conformational change that allows for chloride influx through their central pore into the intracellular compartment of neurons, leading to hyperpolarization of the cell [30, 31]. Like other benzodiazepines, remimazolam can bind to multiple subtypes of the GABA-A receptor but most favorably binds to the alpha-1 subtype [25, 32].

More generally, GABA-A receptor expression on the surface of neurons fluctuates [33]; the density of these receptors may be more permanent at specific synapses, while fluctuations may allow for plasticity in the strength of other inhibitory synapses [34]. GABA-A receptors are expressed ubiquitously in the central nervous system, including the cortex and basal ganglia. However, it is also largely accepted that GABA-A receptor densities vary amongst different subclasses of cells and brain regions [35, 36]. As with all centrally acting anesthetics, it remains to be determined whether the effects of remimazolam are largely attributable to its action within one brain nuclei or more globally by modulating the activity of vast networks of circuitry. Early studies of CNS 7056 conducted in rodent models suggest that the sedating effects were primarily a result of inhibition of signals to and from afferent striatal GABA projections in the substantia nigra pars reticulata. This suppression of the substantia nigra pars reticulata neuronal firing results in loss of the righting reflex and, subsequently, a sedative effect [25]. However, more recent studies suggest that remimazolam likely exerts its effects through a more global influence on neural circuits rather than being confined to specific brain nuclei. These studies suggest that remimazolam's sedative effects may be linked to its modulation of widespread GABAergic activity in the cortex, hippocampus, and thalamus, all of which play crucial roles in memory, awareness, and sleep regulation [37]. Additionally, the rapid and consistent sedative effects observed with remimazolam support the idea of global action, modulating extensive neural circuits rather than being limited to discrete areas [22].

The potential for remimazolam use outside of its current procedural sedation indication is an active area of investigation. More recent work suggests remimazolam also interacts with peripheral receptors in dorsal horn neurons of the spinal cord to promote inhibitory currents [38], potentially peripherally blocking pain transmission. It has also been suggested that remimazolam may regulate the expression of bradykinin receptor B1 as well as its downstream, proinflammatory substrate, Nuclear Factor κB, thereby disrupting neuropathic pain signaling [39]. Nevertheless, remimazolam, like other sedative agents, can be safely combined with analgesics [40] but is unlikely to be sufficient for pain management in the setting of procedural sedation for invasive procedures.

## 3 PHARMACOKINETICS AND PHARMACODYNAMICS

Remimazolam (CNS7056) was developed by the pharmaceutical company PAION AG and has a chemical structure inspired by etomidate and remifentanil. Its design combines elements from existing anesthetic agents, specifically midazolam and remifentanil [19, 41]. The chemical name for remimazolam is methyl 3-[(4S)-8-bromo-1-methyl-6-pyridin-2-yl-4H-imidazo [1,2-a][1,4]benzodiazepin-4-yl]pro-panoate, and it is known by several alternative names such as GW502056, CNS 7056, CNS 7056B, CNS 7056BS, ONO-2745, and HR 7056. Remimazolam's chemical composition includes a benzodiazepine core with a pyridine ring and a methyl group attached to it, making it an ester-based benzodiazepine derivative. The introduction of a carboxylic ester side group into the chemical structure of midazolam is what distinguishes remimazolam and contributes to its unique pharmacokinetic profile [42].

Remimazolam is available in two salt forms: besylate and tosylate. The besylate salt form is designed to improve the stability and solubility of the compound. It is formulated as a lyophilized product, which means it is freeze-dried and requires reconstitution before administration. However, due to its limited solubility in water, larger volumes of remimazolam besylate may be necessary for anesthesia doses [42]. On the other hand, the tosylate salt form of remimazolam was likely chosen strategically to navigate existing patent protections for salts and polymorphic forms. This dual approach in salt forms demonstrates a pharmaceutical strategy aimed at optimizing the drug's properties for clinical use while navigating legal considerations in the pharmaceutical industry.

Remimazolam is administered intravenously (IV). This route of administration allows the drug to quickly reach its target receptors, the GABA-A receptors, leading to rapid onset and short duration of action. In healthy adults receiving doses between 0.01 and 0.3 mg/kg, the maximum sedative effect of remimazolam is achieved within just 3 minutes after injection [43]. The intravenous route ensures precise dosing and control over the drug's effects, making it suitable for use in anesthesia where rapid onset and recovery are crucial [44]. Additionally, remimazolam has been found to be physically compatible with opioid analgesics (remifentanil and fentanyl), muscle relaxants (rocuronium and vecuronium), and other sedatives (dexmedetomidine and midazolam) during simulated Y-site administration, facilitating its use in multimodal anesthesia regimens [45].

The steady-state volume of distribution for remimazolam is approximately 50% of that observed for midazolam. Remimazolam reaches its steady state with similar rapidity to propofol and more quickly compared to midazolam but slightly longer, although likely not clinically meaningfully so, compared to propofol [43, 46-48]. Importantly, remimazolam can reach a steady state following an approximate 2-hour infusion, and this distribution is not significantly influenced by the duration of the infusion [43, 44, 47]. For comparison, midazolam is often estimated to take several days to achieve steady-state concentrations in the body. Remimazolam’s first-order pharmacokinetics provide a wider therapeutic range in which the accumulation and prolonged effects associated with many other hypno-sedating agents can be avoided [47].

Remimazolam undergoes rapid metabolism primarily through tissue esterase hydrolysis by tissue, particularly carboxylesterase 1 (CES1), acting on the carboxylic ester moiety attached to the benzodiazepine core of the drug [25, 49]. Hydrolysis of remimazolam’s carboxylic ester side group by CES1 leads to the formation of an inactive metabolite, known as CNS7054, which has at least 300 times lower affinity for the GABA-A receptors compared to the parent compound CNS7056 [46]. As expected, the tissue-based metabolism is not affected by the presence of renal and hepatic impairment [50] and accounts for the speed at which remimazolam is cleared in comparison to other benzodiazepines. Unlike some drugs whose effects terminate due to redistribution into inactive tissue compartments, remimazolam's clinical effects depend primarily on the speed at which it is broken down by CES1 [51, 52]. The product of this metabolism, a carboxylic acid (CNS-7054), is inactive, providing first-order pharmacokinetic clearance with much greater rapidity than that of midazolam [43, 47]. Thus, this transformation is crucial for the drug's rapid inactivation and termination of its sedative and anesthetic effects.

Remimazolam exhibits rapid systemic clearance, approximately three times greater than midazolam at an equivalent dose of 0.075 mg/kg. This faster clearance contributes to remimazolam's shorter terminal half-life compared to midazolam, with a mean residence time of one-seventh of that of midazolam. Median recovery times after equi-effective doses of remimazolam and midazolam further highlight this rapid elimination, with recovery times of 10 and 40 minutes, respectively [46]. Importantly, clearance is essentially independent of body weight, ensuring consistent elimination rates across different patient weights. Context-sensitive half-times of remimazolam are also notably shorter than those of midazolam, indicating rapid removal from the body after the infusion is stopped [47]. Additionally, depth and duration of sedation in the context of remimazolam alone are dose-dependent [43, 46, 53], as later confirmed with electroencephalogram data, namely the suppression of alpha-band amplitudes [47, 54].

Overall, remimazolam’s low steady-state volume of distribution, in combination with rapid metabolism and systemic clearance, facilitates its swift cessation of effects and elimination from the body, making it an attractive option for situations where rapid recovery from sedation/anesthesia is desired. In short, the growing popularity of remimazolam amongst sedative agents is attributable to its pharmacokinetic and pharmacodynamic profiles: quicker patient recovery times, pharmaco-sparing affects, favorable safety profile, and minimal impact on hemodynamics [47, 48, 55-58]. Such findings are particularly attractive from a hospital administration perspective, wherein time-to-discharge can be delayed with excess sedation [58]. Notably, however, time-to-discharge in patients receiving remimazolam does not differ in a practical manner from those who received midazolam [59].

The safety profile of remimazolam appears promising based on various studies comparing it with other anesthetic agents. A randomized controlled trial comparing remimazolam to propofol during induction and maintenance of anesthesia demonstrated advantages such as less injection pain and lower incidence of hypotension. Additionally, the use of flumazenil in conjunction with remimazolam did not result in adverse events, and the combination was effective in reversing the sedative effects of remimazolam [60]. Another study demonstrated the remimazolam can be safely administered as an IV bolus injection, with dose-dependent effects on mean arterial pressure deemed clinically acceptable [61]. Furthermore, remimazolam showed comparable effects to propofol on cerebral blood flow during carotid endarterectomy in elderly patients, with no significant differences in regional cerebral oxygen saturation or other hemodynamic parameters [62]. Side effects such as bradycardia, hypotension, and respiratory depression may occur; however, the incidence is reduced with remimazolam compared to other sedative agents, including propofol [19]. An additional scoping review demonstrated delayed emergence in 68 of 6740 patients, suggesting some variability in remimazolam metabolism between patients; further, re-sedation and anaphylaxis are rare but potential side effects [63]. Remimazolam's effects on hemodynamics, reduced pain during injection, and reversibility profile appear favorable, collectively suggesting that it is a safe and well-tolerated option for anesthesia.

The standard dose of remimazolam for inducing loss of responsiveness and general anesthesia varies depending on factors such as age and the desired level of sedation. Studies have provided different optimal doses based on various endpoints. For instance, the ED50 and ED90 for loss of responsiveness within two minutes of infusion were found to be 0.07 mg/kg/min and 0.10 mg/kg/min, respectively, and vital signs remained stable during infusion rates of 0.10 mg/kg/min [64]. When compared to propofol, remimazolam IV bolus had success induction-success rates of 89%, 94%, and 100% at doses of 0.2 mg/kg, 0.3 mg/kg, and 0.4 mg/kg, respectively, with lower rates of hypotension and no injection site pain [65].

Remimazolam demonstrates a favorable safety profile for anesthesia use in patients with renal and hepatic impairments based on several studies. In terms of renal impairment, remimazolam's plasma clearance remains comparable to that of healthy subjects, with no accumulation observed in renally impaired individuals [50]. Furthermore, the drug's metabolism, primarily by carboxylesterases, contributes to its suitability for hemodialysis patients without significant renal impact [66]. This contrasts with other short-acting sedatives like midazolam and propofol, which may show accumulation or require dose adjustments in renal impairment cases [67]. Regarding hepatic impairment, the sedative effect and recovery time in severe cases may be slightly increased compared to healthy volunteers, necessitating cautious dosing during maintenance [50, 68]. However, no significant impact was observed with mild or moderate hepatic impairment and dose adjustments are generally not necessary [69].

Remimazolam's interaction with other drugs involves several key considerations. It can be administered alone or in combination with other central nervous system depressants, potentially leading to synergistic or additive effects, necessitating cautious dosing strategies [51]. Opioids have a known synergistic effect with anesthetic induction agents like benzodiazepines, which can influence variables such as time to loss of consciousness, recovery duration, and hemodynamic parameters during anesthesia [61]. This effect highlights the need for appropriate precautions, such as careful monitoring and dosage adjustments, to manage potential interactions when combining remimazolam with other central nervous system depressants.

Remimazolam requires careful consideration for geriatric patients, as age has been identified as a significant covariate. Decreases in ED50/ED95 values for both loss of consciousness and respiratory depression indicate lower doses may be needed to achieve similar effects compared to younger individuals. Optimal bolus doses ranging from 0.25-0.33 mg/kg, 0.19-0.25 mg/kg, and 0.14-0.19 mg/kg have been suggested for patients aged <40, 60-80, and >80 years, respectively. However, there is a sufficient safety margin observed between the onset of loss of consciousness and respiratory depression in all age groups, suggesting that remimazolam can be safely administered to geriatric patients with proper dose adjustments tailored to their age-related responses [61].

Although remimazolam currently lacks specific pediatric labeling, studies have explored its use in pediatric patients. One study involving 418 children with a mean age of 4.6 years used induction doses of 12 mg/kg/h with subsequent infusions of remimazolam at a rate of 1-2 mg/kg/h and intermittent boluses of 0.2 mg/kg. They reported notable hemodynamic changes, with almost half the patients demonstrating a greater than 30% change in mean arterial pressure from baseline and 5% of patients requiring ephedrine. However, they highlighted the rapid recovery benefits of remimazolam, with discharge criteria met within an average of 13.8 minutes after arriving at the post-anesthesia care unit [70]. Although this provides insight, further research into the dosing and effects is warranted for a comprehensive understanding and safe usage of remimazolam in pediatric populations.

## 4 STUDIES REGARDING THE USE OF REMIMAZOLAM FOR PROCEDURAL SEDATION

Several studies have explored the role of remimazolam for procedural sedation, ranging from gastrointestinal procedures to bronchoscopy, pediatric imaging, and sedation in the intensive care unit. A preliminary overview is provided in Table **1**. A summary of specific interventions, sedation-related outcomes, and adverse events is provided in Table **2**.

### 4.1 Gastrointestinal Procedures

Given the expanding world of procedural sedation in gastrointestinal suites, in addition to the rapid turnover for these procedures, the use of remimazolam for procedural sedation has been frequently studied in this setting for a variety of procedures.

In a randomized, controlled, single-center trial by Dong *et al*., the safety and efficacy profiles were compared between remimazolam and propofol when combined with alfentanil for patients undergoing procedural sedation for endoscopic retrograde cholangiopancreatography (ERCP). Patients undergoing ERCP under deep sedation were randomly assigned to either a combination of remimazolam-alfentanil or propofol-alfentanil group. The primary outcome of this study was the prevalence of hypoxia, which was found to be more common in the propofol than the remimazolam group. Analysis of secondary outcomes showed the need for airway maneuvering to be higher in the propofol than remimazolam group. However, the remimazolam group demonstrated longer procedure times, longer sedation times, increased consumption of alfentanil, and increased time to wakefulness. These outcomes were explained by these patients having more complex diseases than the propofol group despite similar indications for ERCP in both groups. The authors concluded that during elective ERCP administration, remimazolam was found to be associated with fewer respiratory events than propofol when administered with alfentanil [71].

In a phase Ib study by Worthington *et al*., the reversal of remimazolam with flumazenil, as well as dose escalation of remimazolam for sedation during colonoscopy, was evaluated. Participants were randomized to the flumazenil reversal group or one of three dose escalation groups. For the flumazenil reversal group, results indicated that after sedation with remimazolam, the time until subjects were fully alert was significantly faster than placebo alone. For the dose escalation groups, the study found that the majority of their subjects were able to complete a colonoscopy with similar amounts of top-up doses and with rapid onset of sedation, short recovery times, and short time to readiness for discharge. Hypoxia was observed in 2 of 6 subjects after administration of a single large dose of remimazolam as part of the flumazenil reversal study and in 9 of 45 subjects during the colonoscopy phase of the study. Hypotension was observed in 24% of subjects in all dose escalation treatment groups. This study was limited by its lack of statistical power and limited inclusion criteria for study participants; however, it serves as a starting point for evaluating remimazolam in procedural sedation for gastrointestinal procedures and assessing dosing [72].

In a phase IIa study by Borkett *et al*., remimazolam was compared with midazolam for sedation in upper gastrointestinal endoscopy. Patients scheduled to undergo diagnostic upper gastrointestinal endoscopy were randomized to receive remimazolam or midazolam. The success of the procedure, sedation level, recovery from sedation, and safety were assessed. Study results indicated that a single medium or high dose of remimazolam resulted in greater procedural success than in the midazolam dose group. The onset of sedation was faster in the remimazolam *vs.* midazolam group. Sedation was maintained with rescue midazolam or propofol as this study was looking only at remimazolam as a single administration. Recovery from sedation was rapid for all groups but notably was influenced by what rescue medication was chosen. No obvious safety differences between remimazolam and midazolam were identified in this study. This study found that a single administration of remimazolam with a dose ranging from 0.10 to 0.20 mg/kg could induce rapid sedation for initiation of upper gastrointestinal endoscopy with a safety profile similar to midazolam. This study concluded that remimazolam exhibited a quick recovery profile. However, limitations of the study included the influence of a choice of rescue medication to maintain sedation for the procedure [55]. In a phase III study by Rex *et al*., remimazolam was compared to placebo and midazolam for procedural sedation use in outpatient colonoscopy. Participants were randomized to receive either remimazolam, placebo, or midazolam. Rescue midazolam was administered to patients in all treatment arms for inadequate sedation at the investigator's discretion. The primary endpoint being measured was meeting three required criteria of colonoscopy completion, no need for rescue medication, and less than a set number of doses to maintain sedation. The primary endpoint was met significantly more in the remimazolam group than midazolam or placebo groups. This study found that patients who received remimazolam required less rescue medication had a faster recovery, and were ready for discharge earlier than patients in the placebo or midazolam groups. The safety profile of remimazolam was comparable to midazolam, with hypoxia occurring with the same frequency in both groups. However, hypotension was less frequent in the remimazolam group [56].

In a meta-analysis of randomized controlled trials by Chang *et al*., the efficacy and safety of remimazolam were compared to propofol for procedural sedation in endoscopy, colonoscopy and hysteroscopy. Twelve randomized controlled trials were included in the meta-analysis. The pooled results demonstrated that patients who received remimazolam, when compared with propofol for procedural sedation, had a lower risk of hypotension, bradycardia, and respiratory depression. No difference was found when remimazolam was compared with propofol for rates of postoperative nausea, vomiting, and dizziness. When compared with propofol, remimazolam was found to have significantly less injection pain. No difference was found in sedation success rate, time to procedural start, time to recovery, or time to discharge when comparing remimazolam to propofol [73].

Overall, studies regarding the use of remimazolam in procedural sedation for gastrointestinal procedures have thus far been favorable. The drug has been compared to commonly used sedative agents propofol and midazolam. This comparison has demonstrated similar safety profiles to midazolam in terms of incidence of hypoxia and hypotension. However, studies have shown that the incidence of hypoxia and hypotension is often significantly decreased when compared to propofol. The drug has shown similar time to onset of procedure as well as recovery with studies that have compared it to midazolam. However, the majority of studies have shown significantly decreased time to initiation of the procedure, faster recovery, and faster time to discharge when compared with propofol. Further studies are needed to elucidate recommended dosing for this procedural setting as well as appropriate adjuncts for analgesia.

### 4.2 Bronchoscopy

In a prospective, double-blinded, randomized, multicenter study by Pastis *et al*., the safety and efficacy of remimazolam were compared with placebo and midazolam for moderate sedation during bronchoscopy. The study found procedural success rates to be greater in the remimazolam arm when compared with the midazolam and placebo arms. Initiation of the procedure was found to be sooner in the remimazolam arm when compared with the midazolam and placebo arms. Time to full alertness at the completion of the procedure was found to be significantly shorter in the remimazolam arm when compared with the midazolam or placebo arms. The conclusions of the study were that remimazolam demonstrated a shorter onset of action and faster neuropsychiatric recovery than midazolam and was found to be both safe and effective for this procedural sedation [74]. A meta-analysis by Zhou *et al*. compared successful sedation between remimazolam *versus* conventional sedatives for patients undergoing bronchoscopy procedures [75]. Although the incidence of successful sedation was similar between both arms, remimazolam provided lower incidences of hypotension and respiratory depression.

### 4.3 Pediatrics

The use of remimazolam for procedural sedation in the pediatric population still requires further review. An initial retrospective study by Hirano *et al*. analyzed patients under 18 years old from 2020 to 2022 who underwent procedural sedation [76]. Despite a continuous infusion of remimazolam, top-up doses of additional sedatives such as ketamine, propofol, and fentanyl were required to achieve adequate sedation. No sentinel adverse events occurred. The study was limited by heterogeneity in procedures, as well as its retrospective nature, without a clear protocol for comparison between sedation modalities.

### 4.4 Intensive Care Unit

The use of remimazolam in the intensive care unit is also of great interest, given that traditional modes of sedation in the intensive care unit involve long context-sensitive half-times (for example, propofol, midazolam, fentanyl, and dexmedetomidine). Remimazolam has been approved in Belgium for the purpose of intensive care unit sedation [19]. A recent randomized pilot study by Tang *et al*. in 2023 demonstrated similar levels of sedation between remimazolam and propofol infusions without significant difference in episodes of hypotension, successful extubation, days without ventilator support, length of stay, or 28-day mortality [77]. The study was limited in its single-center design and small sample size. Further studies are needed to elicit the impact of prolonged remimazolam infusions in the intensive care unit setting.

## 5 OPTIMAL DOSING OF REMIMAZOLAM FOR PROCEDURAL SEDATION

The anesthesiologist carefully titrates the optimal dose of remimazolam based on a patient's comorbidities and procedure being performed. An initial ascending-dose study from Antonik *et al*. was performed to evaluate the dose needed for greater than five minutes of loss of consciousness in the majority of patients. The minimum dose for this effect was found to be 0.075 mg/kg of remimazolam in this cohort of healthy patients, with increased duration of sedation as the dose was increased in subsequent groups [46].

An additional dose-finding study was performed to assess the dosage of remimazolam specifically for colonoscopy in a small study of healthy (American Society of Anesthesiologists Physical Status I) volunteers [72]. Subjects first received a dose of 50mcg of fentanyl, followed five to ten minutes later by remimazolam 0.04, 0.075, or 0.10 mg/kg. If additional doses were needed, subjects were given a repeat dose of 0.04 mg/kg of remimazolam no sooner than two minutes apart, as well as rescue fentanyl if needed. 34/44 subjects were able to complete the colonoscopy using this paradigm.

The same authors studied a separate cohort of six subjects who received a dose of 0.25 mg/kg of remimazolam followed by reversal with 0.5mg of flumazenil or placebo three minutes after initial remimazolam administration. Subjects were fully alert approximately 1 minute after receiving flumazenil compared to 10.5 minutes after placebo [72].

The dose of remimazolam for light and moderate sedation has been explored by Li *et al*. The randomized controlled study enrolled patients undergoing a nerve block procedure and compared remimazolam doses for patients less than 65 years old as well as those aged 65-85. The study determined an optimal dose of 0.08 mg/kg for young patients, while elderly patients had an optimal effect of 0.04 mg/kg [78]. Successful sedation was quantified as light sedation with no requirement for rescue sedation, no moderate or severe pain during the procedure, and no hemodynamic changes over 20% of the patient’s baseline.

Remimazolam has been approved by the United States Food and Drug Administration for intravenous use for procedural sedation for procedures lasting less than or equal to thirty minutes. The current guidelines propose a dose of 5 mg on induction with a maintenance dose of 2.5 mg for adults with at least two minutes between the administration of subsequent doses. This is halved for patients attaining an American Society of Anesthesiologists Physical Status III and IV (2.5 mg induction dose and 1.25 mg maintenance dose) [79].

## 6 PROPOSED ROLE OF REMIMAZOLAM FOR PROCEDURAL SEDATION

There are several characteristics of remimazolam that make it a favorable agent for use in procedural sedation [21]. The first is the safety profile, as the drug has been demonstrated to be associated with a lower incidence of bradycardia, hypotension and respiratory depression when compared to other agents typically utilized for procedural sedation. Remimazolam’s titratability serves as another favorable characteristic for procedural sedation and as previously described, has received United States Food and Drug Administration approval for initial and top-up dosages to maintain sedation. Finally, the ability to utilize flumazenil for reversal of sedation with remimazolam with a return to full alertness in approximately one minute makes it beneficial for procedural sedation. These benefits for procedural sedation have predominantly been described in patients with American Society of Anesthesiologists physical status of I or II; however, remimazolam may have utility at reduced dosages for patients with comorbidities. When considering the use of remimazolam outside of procedural sedation, further studies are required to elucidate the effect of this agent as part of a general anesthetic or as a sole agent intraoperatively for longer procedures.

## CONCLUSION

Remimazolam is a novel benzodiazepine that produces central nervous system inhibition *via* agonism at the GABA-A receptor. This medication exhibits a fast onset and undergoes rapid metabolism in the body *via* hydrolysis by non-specific tissue esterase. Remimazolam is physically compatible with opioid analgesics, muscle relaxants, and other sedatives. Studies have demonstrated the utility of remimazolam in procedural sedation primarily due to its safety profile, titratability, and availability of flumazenil as a reversal agent. Remimazolam has United States Food and Drug Administration approval for intravenous use for procedural sedation for procedures lasting less than or equal to thirty minutes. Guidelines are available to guide initial and top up dosages with recommendations for decreased dosing in patients with comorbidities. Further studies are required to evaluate remimazolam as a sole agent for longer procedures or as an agent used as part of a general anesthetic.

## Figures and Tables

**Table 1:** Studies evaluating remimazolam in clinical use for procedural sedation.

**Study**	**Design**	**Subjects (N)**	**Age (y)**	**ASA Class**	**Procedure**	**Intervention & Dosages**
Dong *et al*. (2023) [71]	Randomized Clinical Trial	518	58-75	I, II, III	Endoscopic retrograde cholangiopancreatography	*Remimazolam group:* Remimazolam infusion 0.2-1 mg/kg/h and alfentanil infusion 0-1 μg/kg/min. *Propofol group:* Propofol bolus 1.5-2 mg/kg followed by propofol infusion 2-6 mg/kg/h and alfentanil infusion 0-1 μg/kg/min.
Worthington *et al*. (2013) [72]	Phase Ib Clinical Trial	45	18-75	I	Colonoscopy	*Flumazenil reversal group:* Remimazolam bolus 0.25 mg/kg followed by flumazenil bolus 0.5 mg or placebo until fully alert. *Dose escalations:* Fentanyl bolus 50 µg followed by remimazolam bolus (0.04, 0.075, or 0.10 mg/kg, depending on the group). Top-up doses 0.04 mg/kg at 2-min intervals.
Borkett *et al*. (2015) [55]	Phase IIa Clinical Trial	100	18-65	I, II	Upper gastrointestinal endoscopy	*Randomized treatment groups:* Single remimazolam bolus 0.10, 0.15, or 0.20 mg/kg; or midazolam 0.075 mg/kg.
Rex *et al*. (2021) [56]	Phase III Clinical Trial	461	19-92	I, II, III	Gastroenterology endoscopy	*All groups:* Fentanyl bolus 50-75 mcg (age-based). *Remimazolam group:* Remimazolam bolus 5 mg. Top up doses 2.5 mg at 2-min intervals (max 5 doses per 15-min). *Placebo group:* Volume equivalent initial bolus and top-up doses. *Midazolam group:* Midazolam bolus 1-1.75 mg (age-based). Top-up doses 0.5-1 mg (max 3 doses per 12-min).
Chang *et al*. 2023 [73]	Meta-analysis	2170	N/A	I, II, III	Gastroenterology endoscopy, colonoscopy, hysteroscopy	*Study selection criteria:* 1. Randomized controlled trials (RCTs). 2. Age > 18 years receiving procedural sedation with propofol or remimazolam. 3. Reported at least one comparative outcome of remimazolam *vs.* propofol.
Pastis *et al*. (2019) [74]	Randomized Clinical Trial	431	50-74	I, II, III	Bronchoscopy	*All groups:* Fentanyl bolus 25-75 μg. *Remimazolam group:* Remimazolam bolus 5 mg. Top-up doses 2.5 mg. *Placebo group:* Volume equivalent initial bolus and top-up doses. *Midazolam group:* Midazolam bolus 1-1.75 mg (age-based). Top-up doses 0.5-1 mg.
Zhou *et al*. (2024) [75]	Meta-analysis	1080	18-75	I, II, III	Bronchoscopy	*Criteria for study selection:* 1. RCT. 2. Procedure type: bendable bronchoscopy. 3. Sedation with remimazolam or conventional sedatives (*i.e*. propofol, midazolam, dexmedetomidine).
Hirano *et al*. (2023) [76]	Pilot study	48	≤ 17	I, II, III	Computerized tomography (CT), Magnetic resonance imaging (MRI), Radiation therapy, Intravenous angiography	Remimazolam infusion 12 mg/kg/h until desired sedation level. Maintenance infusion 1-2 mg/kg/h and boluses 0.2 mg/kg. Top-up doses of fentanyl, ketamine, or propofol were given at the discretion of the clinician.
Tang *et al*. (2023) [77]	Pilot study	60	52-70	I, II, III, IV, V	Sedation for critically ill patients on mechanical ventilation	*Remimazolam group:* Remimazolam infusion 0.3 mg/kg/h, titrated to max 3.0 mg/kg/h until desired sedation level. *Propofol group:* Propofol infusion 3.0 mg/kg/h, titrated to max 12.0 mg/kg/h until desired sedation level.

**Table 2:** Summary of individual trials comparing remimazolam for sedation.

**Study**	**Sedation Related Outcomes**	**Adverse Events**
Dong *et al*. (2023) [71]	Compared to the propofol group, the remimazolam group had: • Longer procedure time: 30 (24.5-42) *vs.* 30 (21-37) minutes, *p =* 0.02 • Longer sedation time: 35 (27.75-45) *vs.* 33 (24-40) minutes, *p =* 0.011 • Greater total alfentanil consumption: 1.55 (1.29-1.97) *vs.* 1.38 (1.18-1.77), *p <* 0.001 • Longer awake time: 8 (6-10) *vs.* 7 (5-9) minutes, *p <* 0.001 • Longer recovery time: 12 (9-13.25) *vs.* 10 (8-12) minutes, *p <* 0.001	Compared to the propofol group, the remimazolam group showed: • Lower incidence of hypoxia: 9.6% *vs.* 15.7%, *p =* 0.04 • Less need for airway maneuvering due to hypoxia, *p =* 0.04 • Lower incidence of hypotension, *p <* 0.001
Worthington *et al*. (2013) [72]	Dose escalation group successful colonoscopies: • 10/15 (0.04 mg/kg), 15/15 (0.075 mg/kg), and 9/14 (0.10 mg/kg) • Mean number of remimazolam top-up doses was 5.2-5.4 across groups (mean cumulative doses of 0.26-0.31 mg/kg) Dose escalation group unsuccessful colonoscopies: • 8 had insufficient initial sedation (5 in 0.04 mg/kg, 3 in 0.10 mg/kg groups) • 2 had due to inability to maintain sedation Flumazenil reversal group median time to full alertness was 1.0 minutes *vs.* placebo 10.5 minutes.	• Hypotension: 24% of subjects across all dose escalation groups: 3 (0.04 mg/kg), 5 (0.075 mg/kg), and 3 (0.10 mg/kg) subjects • Hypoxia was observed in 1 (0.04 mg/kg), 5 (0.075 mg/kg), and 3 (0.10 mg/kg) subjects during dose escalation. • One subject (0.075 mg/kg group) required oxygen *via* facemask. • Hypoxia occurred in 2/6 subjects during flumazenil reversal (0.25 mg/kg dose) and 9/45 subjects during colonoscopy.
Borkett *et al*. (2015) [55]	Procedure success rates with a single dose were: • Remimazolam: 32% (0.10 mg/kg), 56% (0.15 mg/kg), 64% (0.20 mg/kg) • Midazolam: 44% (0.075 mg/kg) Percentage of patients with no procedural recall: • Remimazolam: 76% (0.10 mg/kg), 84% (0.15 mg/kg), 80% (0.20 mg/kg) • Midazolam: 80%	• Hypotension: 0% for all remimazolam doses; 4% for midazolam • Hypoxia (SpO_2_ <90%): 16%, 20%, 24% for remimazolam (0.10, 0.15, 0.20 mg/kg); 20% for midazolam • Airway intervention without O_2_: 4%, 12%, 12% for remimazolam (0.10, 0.15, 0.20 mg/kg); 8% for midazolam • Airway intervention with O_2_: 12%, 0%, 4% for remimazolam (0.10, 0.15, 0.20 mg/kg); 0% for midazolam
Rex *et al*. (2021) [56]	• Procedural success rates: remimazolam 91.3%, placebo 1.7%, midazolam 25.2% (*p <* 0.0001 for remimazolam *vs.* placebo) • Rescue medication not needed: remimazolam 96.6%, placebo 5.0%, midazolam 35.9% (*p <* 0.0001 for remimazolam *vs.* placebo)	• Treatment emergent adverse events: remimazolam 73.6%, placebo 78.3%, midazolam 91.2% • Hypotension: remimazolam 38.9%, placebo 41.7%, midazolam 61.8% • Hypoxia: remimazolam 1.0%, placebo 3.3%, midazolam 1.0%
Chang *et al*. (2023) [73]	• Sedation success rates were similar between remimazolam and propofol groups (OR 1.40, 95% CI 0.41-4.76). • No significant differences were found in time to loss of consciousness, recovery, or discharge between the two groups.	Compared to the propofol group, patients receiving remimazolam had a lower risk of: • Bradycardia (OR 0.28, 95% CI 0.14-0.57) • Hypotension (OR 0.26, 95% CI 0.22-0.32) • Respiratory depression (OR 0.22, 95% CI 0.14-0.36)
Pastis *et al*. (2019) [74]	• Procedure success rates: remimazolam 80.6%, placebo 4.8%, midazolam 32.9% (*p <* 0.0001) • No rescue sedation needed: remimazolam 84.2%, placebo 9.5%, midazolam 46.6% (*p <* 0.0001) • Median time to full alertness: remimazolam 6.0 min, placebo 13.6 min, midazolam 12.0 min (*p =* 0.0001)	• Hypoxia: remimazolam 21.8%, placebo 20.3%, midazolam 18.8% (*p =* 0.864) • Hypotension: remimazolam 41.9%, placebo 62.7%, midazolam 49.3% (*p =* 0.004) • Hypertension: remimazolam 61.4%, placebo 52.5%, midazolam 59.4% (*p =* 0.245)
Zhou *et al*. (2024) [75]	• Remimazolam achieved successful sedation in 473/538 patients (87.9%) compared to 245/301 (81.4%) with conventional sedatives. • Meta-analysis showed no significant difference in sedation success rates between remimazolam and conventional sedatives (RR 1.35, *p =* 0.474).	Compared to the conventional sedative, patients receiving remimazolam had lower rates of: • Hypotension (RR 0.61, *p =* 0.027) • Respiratory depression (RR 0.50, *p =* 0.002) No significant differences were found for other adverse effects, including hypertension, hypoxemia, bradycardia, tachycardia, and injection pain.
Hirano *et al*. (2023) [76]	• 95% of patients received remimazolam in conjunction with other sedatives or analgesics (ketamine, propofol, or fentanyl).	• No sentinel adverse events were recorded. • Mean arterial pressure (MAP) decreased in 93% of patients, with a median drop of 22.6% from baseline (range: -58.2% to +19.2%). • Significant MAP fluctuations were common: 88.4% of patients experienced ≥20% change, and 62.8% experienced ≥30% change from baseline (increase or decrease).
Tang *et al*. (2023) [77]	• Target deep sedation (RASS -4 or -5) was achieved in 94.1% of assessments with remimazolam and 95.5% with propofol. Rescue sedation use was similar between groups.	• No significant differences were found in ventilator-free hours, extubation success, ICU stay, or 28-day mortality. • Hypotension occurred in 53.3% of remimazolam patients and 60.0% of propofol patients, with no statistically significant difference between groups.

## LIST OF ABBREVIATIONS

CES1: Carboxylesterase 1
CT: Computerized Tomography
ERCP: Endoscopic Retrograde Cholangiopancreatography
GABA: Gamma-aminobutyric Acid
IV: Intravenously
MRI: Magnetic Resonance Imaging
